# Comparison of Ultrasound-Guided Subcostal Transversus Abdominis Plane Block and Quadratus Lumborum Block in Laparoscopic Cholecystectomy: A Prospective, Randomized, Controlled Clinical Study

**DOI:** 10.1155/2019/2815301

**Published:** 2019-02-03

**Authors:** Çağdaş Baytar, Canan Yılmaz, Derya Karasu, Serra Topal

**Affiliations:** ^1^Erciş State Hospital, Department of Anesthesiology and Reanimation, Van, Turkey; ^2^Health Sciences University, Bursa Yüksek Ihtisas Training and Education Hospital, Department of Anesthesiology and Reanimation, Bursa, Turkey

## Abstract

**Background:**

The aim of this study was to compare the effectiveness of ultrasound-guided (USG) subcostal transversus abdominis plane (TAP) block and quadratus lumborum (QL) block as preventive analgesia methods after laparoscopic cholecystectomy.

**Methods:**

A total of 120 patients, 18–75 years of age, were separated into 2 groups preoperatively. Patients in group TAP (*n* = 60) received 0.3 ml/kg bupivacaine with USG bilateral subcostal TAP block; patients in group QL (*n* = 60) received 0.3 ml/kg bupivacaine with USG bilateral QL block. Patients were assessed 24 h postoperatively, and pain scores, time to first analgesia requirement, total analgesia dose, and postoperative complications during the first 24 h were recorded.

**Results:**

Fifty-three patients in group TAP and 54 in group QL were ultimately evaluated. No statistically significant difference was found in at rest and dynamic visual analog scale scores between the groups. There was also no statistically significant difference between the groups with regard to total analgesia consumption. Although the duration of anesthesia was significantly longer in group QL, no statistically significant difference was found in the duration of surgery between the groups (*p* < 0.05).

**Conclusions:**

Results of this study demonstrated that USG subcostal TAP and QL blocks similarly reduced postoperative pain scores and analgesia consumption, with high patient satisfaction. However, subcostal TAP block could be considered preferable to QL block because it can be applied easily and in a shorter time.

## 1. Introduction

Despite advances in surgical techniques and anesthesia management, postoperative pain remains an important issue. Pain that develops after surgical intervention is multifactorial, the severity of which varies according to factors including the extent of surgical trauma, anesthesia technique, and the physiological, psychological, emotional, and sociocultural characteristics of the patient [1]. The aims of treating postoperative pain are to eliminate or, at least, reduce pain to a minimum level, to accelerate the healing process, and to avoid side effects that can emerge with treatment. Nonopioid systemic analgesics, such as nonsteroid anti-inflammatory drugs (NSAIDs), antidepressants, and alpha-2 agonists, are used for preventive analgesia instead of opioids in some cases, and sometimes can be used as a part of a multimodal analgesia regimen, especially together with opioids [2, 3]. Intraoperatively, lidocaine in the form of bolus or infusion, and preoperative gabapentin or pregabalin, can be used for preventive analgesia [4, 5]. Together with the increasing use of ultrasound (US), various truncal blocks are performed under US guidance to eliminate postoperative pain and reduce the need for opioids in patients undergoing laparoscopic cholecystectomy.

The aim of the present study was to compare the efficacy of subcostal transversus abdominis plane (TAP) and quadratus lumborum (QL) blocks applied under US guidance as methods of preventive analgesia in laparoscopic cholecystectomy operations.

## 2. Methods

### 2.1. Study Design

The study protocol was approved by the Local Ethics Committee and registered with the Australian New Zealand Clinical Trials Registry (Ref: ACTRN12617000891325). Written informed consent was obtained from each patient, and the study was conducted in accordance with the principles of the Declaration of Helsinki. This was a single-center, prospective, randomized, controlled, and double-blinded study. Patients and postoperative evaluations were blinded to the study. The investigation included patients aged 18–75 years with American Society of Anesthesiologists (ASA) score I-II, who were scheduled to undergo elective laparoscopic cholecystectomy. Patients were excluded from the study if they had impaired bleeding diathesis, a mental disorder, history of laparotomy, allergy to the drugs used, renal failure patients, infection in the area where the block was to be applied, body mass index (BMI) > 35 kg/m^2^, or inability to communicate. Patients who were discharged before 24 h had block failure (lack of sensorial block in the pinprick test performed postoperatively in the recovery room) or a problem with patient-controlled analgesia (PCA) devices (running out of battery, removal by the patient, and removal by nurse) were excluded. The patients were randomly assigned to 1 of 2 groups using the sealed envelope technique: TAP (*n* = 60) and QL (*n* = 60).

### 2.2. Anesthesia Management

Premedication was administered to all patients (0.01–0.02 mg/kg IV midazolam; Zolamid, Defarma, Ankara, Turkey), and routine monitoring was performed. Following anesthesia induction using 1-2 mcg/kg fentanyl (Talinat, Vem, Istanbul, Turkey), 2-3 mg/kg propofol (propofol 2%, Fresenius, Fresenius Kabi, Bad Homborg, Germany), and 0.6 mg/kg rocuronium (Curon, Mustafa Nevzat, Istanbul, Turkey), the patients were intubated. Low-flow sevoflurane (Sevorane Likit 100%, AbbVie, Queenborough Kent, England) was applied as maintenance anesthesia at a rate of 1 L/min in a 50% air/50% oxygen mixture with a minimum alveolar concentration of 1. Intraoperatively, additional drug doses were administered as needed.

### 2.3. TAP Block Procedure

In group TAP (*n* = 60), the TAP block was performed after intubation with the patient in the supine position by the same experienced anesthesiologist. The USG (Esaote, MyLab30Gold Cardiovascular, Florence, Italy) probe was placed on the upper abdominal wall obliquely along the subcostal edge close to the midline. After identification of the rectus abdominis muscle (RAM), by shifting the probe obliquely along the subcostal line toward the lateral, the transverse abdominis muscle (TAM) was located below the RAM. With the USG probe, an 80 mm, 22-gauge block needle (Stimuplex Ultra, B Braun, Melsungen AG, Germany) was placed between the RAM and TAM in the same plane (in-plane technique).

A preprepared solution of 0.3 ml/kg 0.25% bupivacaine (max. 20 ml) (Buvasin, Vem, Istanbul, Turkey) was injected bilaterally between these two muscles and was observed to spread toward the lateral side of the RAM. After completion of the TAP block, surgery was commenced.

### 2.4. QL Block Procedure

In group QL (*n* = 60), the QL-2 block was performed after intubation with the patient in the lateral decubitus position by the same experienced anesthesiologist. The USG probe was placed on the anterior axillary line at the level of the umbilicus, the abdominal muscles were visualized, and the probe was advanced toward the posterior. After visualization of the QL muscle, the probe was fixed by determining the intersection with the transverse fascia in the anterior and lateral side of the muscle. By advancing the block needle with the USG probe to be in the same plane (in-plane technique), the point between the QL muscle and the middle thoracolumbar fascia was reached. A preprepared solution of 0.3 ml/kg 0.25% bupivacaine max 20 ml was administered bilaterally. After completion of the QL block, surgery was commenced.

### 2.5. Analgesia Management

Ten minutes before the end of the operation, all patients were administered intravenous 20 mg tenoxicam (Tilcotil, Deva, Istanbul, Turkey), 10 mg metoclopramide (Metpamid, Recordati, Istanbul, Turkey), and 50 mg ranitidine (Ulcuran, Yavuz, Istanbul, Turkey). For postoperative pain control, an intravenous PCA device (CADD-Legacy PCA, Smiths Medical, St Paul, MN, USA) was prepared with an intravenous solution of 54 ml saline + 6 ml tramadol (300 mg) (Tramosel, Haver, Istanbul, Turkey). The bolus dose was set to 5 ml, with a lock-out time of 30 min and no basal infusion. The first bolus dose was administered when the visual analog scale (VAS) score was >3.

### 2.6. Outcomes

Primary outcomes included pain scores and consumption of rescue analgesics. Pain was evaluated according to resting VAS and dynamic VAS (DVAS) scores. Another anesthesiologist evaluated VAS scores at 0, 1, 6, 12, and 24 h. Also, tramadol consumption during 24 hours and the use of another rescue analgesia (when VAS >5, 1 gr paracetamol was ordered to be given) were recorded by the same anesthesiologist. Secondary outcomes included hemodynamic parameters (blood pressure >140/90 mmHg: hypertension, blood pressure <90/60 mmHg: hypotension, heart rate >100/min: tachycardia, heart rate <60/min: bradycardia), side effects (agitation, speech difficulties, drowsiness, mental changes, tinnitus, dizziness, tremors, and numbness), and patient and surgeon satisfactions (very satisfied, satisfied, undecided, and not satisfied).

### 2.7. Statistical Analysis

To define the size of the sample, a pilot study was performed, taking a power of 0.80 and confidence interval of 0.95 as reference and, as a result, the minimum sample size required was 84 individuals. For statistical evaluation, SPSS version 22 (IBM Corporation, Armonk, NY, USA) for Windows (Microsoft Corporation, Redmond, WA, USA) was used. In the descriptive statistics, quantitative data are expressed as mean ± standard deviation (SD) and qualitative data are expressed as percentage (%) values. Conformity to normal distribution was assessed using the Kolmogorov–Smirnov test. For data not exhibiting normal distribution, the Mann–Whitney *U* test, Kruskal–Wallis test, and the chi-squared tests were used; *p* < 0.05 was considered to be statistically significant.

## 3. Results

**Figure 1:**
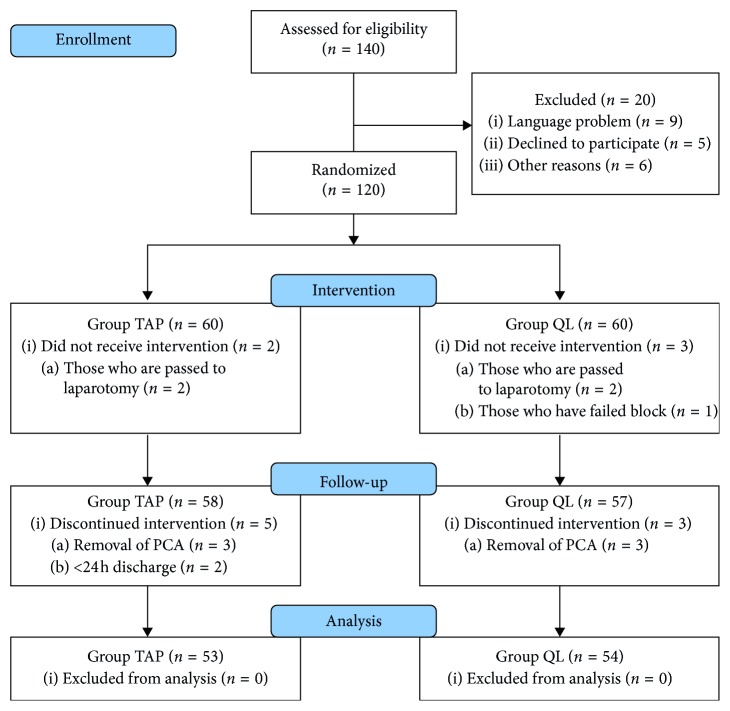
Trial flow diagram.

Of 140 patients who underwent laparoscopic cholecystectomy, 120 were included in the study and 107 were evaluated statistically (Figure 1) (6 patients of PCA removal before 24 hours, 4 patients due to passing to laparotomy, 2 patients because of discharging before 24 hours, and 1 patient due to block failure were excluded from the study). Demographic data are summarized in Table 1. The duration of anesthesia was significantly longer in group QL (*p*=0.013).

### 3.1. Primary Outcomes

There was no statistically significant difference in VAS and DVAS scores between patients in group TAP and group QL at 0, 1, 6, 12, and 24 h (*p* > 0.05) (Tables 2 and 3). No statistically significant difference was found between the groups with regard to intraoperative opioid consumption (group TAP, 1.63 ± 0.23; group QL, 1.67 ± 0.27; *p* > 0.05).

The patients were followed up for a period of 24 h with regard to PCA device use. No statistically significant difference was found in the number of analgesia doses delivered by the PCA device and the total amount of tramadol consumed between group TAP and group QL (Table 4). There was no requirement for additional analgesia postoperatively.

### 3.2. Secondary Outcomes

No statistically significant difference was found between the groups with regard to heart rate and mean blood pressure (*p* > 0.05). No statistically significant difference was found in intraoperative complications (hypertension, *p*=1.000; hypotension, *p*=0.440; bradycardia, *p*=0.278; tachycardia, *p*=1.000) (*p* > 0.05) between the groups. In the comparison of postoperative complications, nausea and vomiting was recorded in 3 patients in group TAP and 2 patients in group QL, and hypertension was observed in 1 patient in group QL. Patient satisfaction was reported as very satisfied by 47, satisfied by 4, and undecided by 2 of the 53 patients in group TAP and very satisfied by 48, satisfied by 3, and undecided by 3 of the 54 patients in group QL. No statistically significant difference with regard to patient and surgeon satisfaction was found between the groups (*p* > 0.05).

## 4. Discussion

This prospective, randomized, double-blinded study compared TAP block and QL block for preventive analgesia in patients who underwent laparoscopic cholecystectomy under general anesthesia. The intraoperative consumption of fentanyl was similar in both groups. No significant difference was found in VAS and DVAS scores between the groups, nor was there any difference in the 24 h consumption of tramadol.

While there was no significant difference with regard to operating time between the groups, duration of anesthesia in group QL was statistically longer (*p* < 0.05). Duration of anesthesia in group QL was approximately 8 minutes longer than group TAP. The QL block is a deep regional block and has to be performed in lateral decubitus position. The QL block application requires more experience and knowledge of sonoanatomy. These factors explain the difference in duration of anesthesia.

Oksar et al. [6] compared subcostal TAP block under US guidance, classic TAP block, and a control group (intravenous tramadol PCA) in patients who underwent cholecystectomy. Postoperative analgesia consumption was higher in the control group, and the VAS scores in the subcostal block group were found to be lower than those of the other two groups. In a study by Bhatia et al. [7], classic TAP block under US guidance, subcostal TAP block, and a control group were compared for postoperative analgesia. The subcostal TAP block was determined to have provided more effective postoperative analgesia in the first 24 h than both the classic TAP block and the control group in laparoscopic cholecystectomies. In another observational study, subcostal TAP block was demonstrated to provide effective postoperative analgesia for the first 24 h in patients undergoing upper abdominal surgery [8].

In a study by Ma et al. [9], which investigated the regional analgesic efficacy of subcostal TAP block applied under US guidance, local anesthetic was administered unilaterally to the patients. Throughout the first postoperative 12 h, patients were examined at intervals using pinprick and loss-of-heat sensitivity tests. Subcostal TAP block was reported to have provided effective and long-lasting analgesia in the anterior abdominal wall, from the xiphoid to the edge of the lower abdomen. Because port entries in laparoscopic cholecystectomies are located above the umbilicus, bilateral subcostal TAP block was preferred in the current study and effective postoperative analgesia was achieved.

In a randomized controlled study involving 70 patients who underwent laparoscopic gynecological surgery, QLB-2 was applied under US guidance, which reduced resting and dynamic postoperative pain compared with the control group [10]. Kadam [11] reported the use of the QL block technique under US guidance as the postoperative analgesia technique in a laparotomy case and recommended QL block for major abdominal surgeries. It was also reported that there was a need for more extensive, randomized, controlled studies to compare this technique with the TAP block. In the current, prospective, randomized study, QL-2 block and subcostal TAP block applied under US guidance were compared in laparoscopic cholecystectomies.

In a randomized controlled study involving 76 patients, Blanco et al. [12] compared QL block and classic TAP block for postoperative pain following caesarean section. All patients received 1 g of paracetamol orally every 6 hours and 50 mg of diclofenac every 8 hours. While postoperative morphine consumption was found to be higher in the TAP block patients, no significant difference was found between the two groups with regard to VAS scores. The fact there was no difference in the VAS scores could be associated with higher postoperative opioid consumption in the TAP group. In the current study, no significant difference was found between the QL block and subcostal TAP block groups with regard to VAS scores or opioid consumption. Although QL block is superior to TAP block for postoperative analgesia in caesarean section, subcostal TAP block is effective as QL block for postoperative analgesia in laparoscopic cholecystectomy. Blanco et al. [12] reported similar values in both groups for respiratory rate, blood pressure, heart rate, and oxygen saturation. In the current study, no difference was found between the groups with regard to intraoperative hemodynamic parameters.

In a randomized, controlled study involving 50 patients, in the QL block applied for postoperative pain following caesarean section operation, one-half of the patients were administered saline and the other half was administered 0.125% bupivacaine [13]. In the first postoperative 24 h, the VAS and DVAS scores of the group administered local anesthetic were found to be significantly lower and the postoperative morphine consumption in the same group was also found to be significantly low. However, it was reported that, despite the efficacy of QL block in postoperative analgesia, the operator should be experienced in US and have extensive sonoanatomy knowledge. The reason that the duration of anesthesia in the QL block in the current study was found to be statistically significantly longer could be that better sonoanatomy knowledge is required for QL block compared with TAP block and, therefore, there were difficulties in application.

There were some limitations to the present study. Laparoscopic cholecystectomy is minimal invasive surgery, so there seems to be less postoperative pain found than that in laparotomy. But, as stated in the literature [14], laparoscopic cholecystectomy is a surgical procedure that frequently results in significant immediate postoperative pain and the need for rescue analgesia in the PACU. Furthermore, the depth of anesthesia was not monitored, which could be considered an additional limitation.

## 5. Conclusion

The results of the current study demonstrated that subcostal TAP and QL blocks under general anesthesia before the surgical incision in laparoscopic cholecystectomies reduced postoperative VAS and DVAS scores and tramadol consumption to a similar level. In upper abdominal surgery, such as laparoscopic cholecystectomy, subcostal TAP block applied under US guidance can be considered to have the advantages of easier application and a shorter time compared with QL block.

## Figures and Tables

**Table 1:** Demographic and clinical data.

	Group TAP (*n* = 53)	Group QL (*n* = 54)	*p*
Age (years)^#^	48.12 ± 12.42	46.42 ± 16.57	0.393
Sex^#^ (male/female)	11/42	15/39	0.534
Height^#^ (cm)	164.66 ± 7.79	165.33 ± 8.20	0.564
Weight^#^ (kg)	78.05 ± 13.25	75.35 ± 10.23	0.249
BMI^#^ (kg/m^2^)	28.79 ± 4.87	27.72 ± 4.08	0.326
ASA (I/II)	12/41	19/35	0.224

ASA: American Society of Anesthesiologists, BMI: body mass index, TAP: transversus abdominis plane, and QL: quadratus lumborum. ^#^Mean ± standard deviation, *n* (%).

**Table 2:** Fentanyl consumption, duration of surgical procedure, and anesthesia (min).

	Group TAP (*n* = 53)	Group QL (*n* = 54)	*p*
Fentanyl^#^(mcg/kg)	1.63 ± 0.23	1.67 ± 0.27	0.056
Duration of surgical procedure^#^ (min)	49.90 ± 16.79	51.22 ± 17.24	0.732
Duration of anesthesia (min)	58.16 ± 16.76	66.40 ± 20.35	0.013^*∗*^

TAP: transversus abdominis plane; QL: quadratus lumborum. ^#^Mean ± standard deviation. ^*∗*^*p* < 0.05.

**Table 3:** Comparison of patient-controlled analgesia in groups.

PCA	Group TAP (*n* = 53)	Group QL (*n* = 54)	*p*
First analgesic time^#^ (min)	63.73 ± 103.42	70.00 ± 92.76	0.187
Tramadol consumption (mg)	83.43 ± 71.25	86.66 ± 67.82	0.754

PCA: patient-controlled analgesia, TAP: transversus abdominis plane, and QL: quadratus lumborum. ^#^Mean + standard deviation, *n* (%).

**Table 4:** VAS and DVAS scores.

Time (hour)	VAS/DVAS	Group TAP (*n* = 53)	Group QL (*n* = 54)	*p*
0^#^	VAS	1.33 ± 1.50	1.03 ± 1.18	0.418
	DVAS	2.42 ± 2.10	2.42 ± 2.10	0.879

1^#^	VAS	0.75 ± 1.29	0.70 ± 0.76	0.227
	DVAS	1.94 ± 1.87	1.90 ± 1.49	0.720

6^#^	VAS	0.47 ± 0.95	0.42 ± 0.74	0.734
	DVAS	1.38 ± 1.52	1.50 ± 1.38	0.535

12^#^	VAS	0.20 ± 0.53	0.22 ± 0.46	0.554
	DVAS	1.00 ± 1.28	0.92 ± 1.24	0.815

24^#^	VAS	0.11 ± 0.37	0.09 ± 0.29	0.951
	DVAS	0.32 ± 0.85	0.46 ± 0.88	0.402

TAP: transversus abdominis plane, QL: quadratus lumborum, VAS: visual analog scale, and DVAS: dynamic visual analog scale. ^#^Mean + standard deviation.

## Data Availability

The data used to support the findings of this study are available from the corresponding author upon request.

## References

[1] Bayar M., İlhan Y., Önal A., Akkuş M., Çifter Ç. (1998). Laparoskopik kolesistektomilerde intraperitoneal bupivakain uygulamasının postoperatif ağrı ve katekolamin düzeylerine etkileri. *Ağrı Dergisi*.

[2] Trabulsi E. J., Patel J., Viscusi E. R., Gomella L. G., Lallas C. D. (2010). Preemptive multimodal pain regimen reduces opioid analgesia for patients undergoing robotic-assisted laparoscopic radical prostatectomy. *Urology*.

[3] Tauzin-Fin P., Bernard O., Sesay M. (2014). Benefits of intravenous lidocaine on post-operative pain and acute rehabilitation after laparoscopic nephrectomy. *Journal of Anaesthesiology Clinical Pharmacology*.

[4] Sztark R. K., Paul J. E. (2006). Preoperative gabapentin for postoperative analgesia: a meta-analysis. *Canadian Journal of Anesthesia*.

[5] Aubrun F., Mazoit J.-X., Riou B. (2012). Postoperative intravenous morphine titration. *British Journal of Anaesthesia*.

[6] Oksar M., Koyuncu O., Turhanoglu S., Temiz M., Oran M. C. (2016). Transversus abdominis plane block as a component of multimodal analgesia for laparoscopic cholecystectomy. *Journal of Clinical Anesthesia*.

[7] Bhatia N., Arora S., Jyotsna W., Kaur G. (2014). Comparison of posterior and subcostal approaches to ultrasound-guided transverse abdominis plane block for postoperative analgesia in laparoscopic cholecystectomy. *Journal of Clinical Anesthesia*.

[8] Mukherjee A., Guhabiswas R., Kshirsagar S., Rupert E. (2016). Ultrasound guided oblique subcostal transversus abdominis plane block: an observational study on a new and promising analgesic technique. *Indian Journal of Anaesthesia*.

[9] Ma J., Jiang Y., Tang S. (2017). Analgesic efficacy of ultrasound-guided subcostal transversus abdominis plane block. *Medicine*.

[10] Ishio J., Komasawa N., Kido H., Minami T. (2017). Evaluation of ultrasound-guided posterior quadratus lumborum block for postoperative analgesia after laparoscopic gynecologic surgery. *Journal of Clinical Anesthesia*.

[11] Kadam V. (2013). Ultrasound-guided quadratus lumborum block as a postoperative analgesic technique for laparotomy. *Journal of Anaesthesiology Clinical Pharmacology*.

[12] Blanco R., Ansari T., Riad W., Shetty N. (2016). Quadratus lumborum block versus transversus abdominis plane block for postoperative pain after cesarean delivery. *Regional Anesthesia and Pain Medicine*.

[13] Blanco R., Ansari T., Girgis E. (2015). Quadratus lumborum block for postoperative pain after caesarean section. *European Journal of Anaesthesiology*.

[14] Szental J. A., Webb A., Weeraratne C., Campbell A., Sivakumar H., Leong S. (2014). Postoperative pain after laparoscopic cholecystectomy is not reduced by intraoperative analgesia guided by analgesia nociception index (ANI®) monitoring: a randomized clinical trial. *British Journal of Anaesthesia*.

